# Urinary Markers of Tubular Injury in Early Diabetic Nephropathy

**DOI:** 10.1155/2016/4647685

**Published:** 2016-05-16

**Authors:** Temesgen Fiseha, Zemenu Tamir

**Affiliations:** Department of Clinical Laboratory Science, College of Medicine and Health Sciences, Wollo University, Dessie, Ethiopia

## Abstract

Diabetic nephropathy (DN) is a common and serious complication of diabetes associated with adverse outcomes of renal failure, cardiovascular disease, and premature mortality. Early and accurate identification of DN is therefore of critical importance to improve patient outcomes. Albuminuria, a marker of glomerular involvement in early renal damage, cannot always detect early DN. Thus, more sensitive and specific markers in addition to albuminuria are needed to predict the early onset and progression of DN. Tubular injury, as shown by the detection of tubular injury markers in the urine, is a critical component of the early course of DN. These urinary tubular markers may increase in diabetic patients, even before diagnosis of microalbuminuria representing early markers of normoalbuminuric DN. In this review we summarized some new and important urinary markers of tubular injury, such as neutrophil gelatinase associated lipocalin (NGAL), kidney injury molecule-1 (KIM-1), liver-type fatty acid binding protein (L-FABP), N-acetyl-beta-glucosaminidase (NAG), alpha-1 microglobulin (A1M), beta 2-microglobulin (B2-M), and retinol binding protein (RBP) associated with early DN.

## 1. Introduction

Diabetic nephropathy (DN) is a common and serious complication of diabetes associated with adverse outcomes of renal failure, cardiovascular disease, and premature mortality [1–3]. It is also the leading cause of end-stage renal disease (ESRD), requiring costly renal replacement therapy in the form of dialysis or transplantation [4]. Early and accurate identification of DN is therefore of critical importance to improve clinical outcomes. Clinically, the appearance of pathological albuminuria, microalbuminuria, is considered a hallmark of early onset of DN. However, a substantial proportion of renal impairment occurs among normoalbuminuric diabetic patients and is associated with more advanced diabetic glomerular lesions and increased risk of progression [5–7]. Moreover, since microalbuminuria is diagnosed once significant glomerular damage has occurred, changes in albuminuria are being increasingly recognized as complementary rather than obligatory manifestations of DN [8, 9].

Recently, changes in the renal tubules, which may be termed diabetic tubulopathy, are increasingly implicated in the development of progressive diabetic kidney disease [10, 11]. It has been reported that, in addition to the glomeruli, the renal tubules are heavily involved in the pathogenesis of DN [12, 13]. In line with this, several glomerular and tubular biomarkers predicting onset or progression of DN have been identified and are becoming increasingly important in clinical diagnostics. The urinary concentrations of these damage markers (both glomerular and tubular) are elevated in diabetic patients and are associated with the severity of DN [14]. Interestingly, some of these markers already are elevated in normoalbuminuric diabetic patients with normal estimated glomerular filtration rate (eGFR) [14]. Therefore, more sensitive and specific markers in addition to albuminuria are needed to predict the development and progression of DN in diabetics even at the very early stage (normoalbuminuric DN).

Tubular injury, as shown by the detection of tubular damage markers in the urine, is a critical component of the early course of DN and has been suggested to contribute in a primary way, rather than a secondary manner, to the development of early DN [15, 16]. Urinary excretion of these tubular markers could, therefore, be useful for assessing an initial malfunction or damage of the renal tubules in the early stage of potentially progressive DN. Several tubular damage markers recently have been discovered and have clinical implications as markers for the development and progression of DN. Some studies have demonstrated that these tubular damage markers increase in patients with diabetes, even before diagnosis of microalbuminuria representing early markers of normoalbuminuric DN with a good sensitivity and specificity [17–19]. This review article summarizes some new and important urinary markers of tubular injury associated with early DN.

## 2. Neutrophil Gelatinase Associated Lipocalin

Neutrophil gelatinase associated lipocalin (NGAL) is a small, 25-kDa, protein that belongs to the lipocalin protein family released from neutrophils and many epithelial cell types including kidney tubular cells. It is representative of the functioning tubular mass and produced as a response to tubular injury [20]. Urinary NGAL levels were found to be markedly elevated in patients with diabetes when compared with nondiabetic control subjects [14, 21–27] and to correlate negatively with eGFR and positively with serum Cystatin C (CysC), serum creatinine (SCr), proteinuria, albuminuria, and urinary albumin excretion (UAE) and albumin-creatinine ratio (UACR), indicating the possible clinical application of urinary NGAL as a complementary marker for early detection of DN [19, 25–29]. It is also significantly correlated with the duration of diabetes, glycemic control (HbA1c), and urinary interleukin-18 (IL-18: proinflammatory biomarker) and angiotensinogen (renin-angiotensin system biomarker), suggesting urinary NGAL as a useful noninvasive tool for the evaluation of renal involvement in diabetes [21, 23, 25, 28, 29].

In diabetics, urinary excretion of NGAL was significantly higher in microalbuminuric in comparison with normoalbuminuric patients and controls and correlated positively with UACR, indicating diabetic tubular damage at the early stage of DN [18, 23, 25, 26, 30]. Urine levels of NGAL were significantly higher in microalbuminuria group compared to normoalbuminuria and were positively correlated to UACR in both diabetes and prediabetes, which suggested that tubular damage may play major role in the development of nephropathy in prediabetes [30]. It was also suggested that NGAL might play an important role in the pathophysiology of renal adaptation to diabetes, and its measurement might become a useful and noninvasive tool for the evaluation of renal involvement in these patients as well as for the early diagnosis of incipient DN [26]. In addition, urinary NGAL showed better area under curve (AUC; diagnostic accuracy) for estimating microalbuminuria, demonstrating its value as a more suitable and sensitive marker for detecting the onset of DN [25, 30].

Higher urinary levels of NGAL have been found in diabetics compared to controls, even in normoalbuminuric patients, indicating that diabetic tubular damage may develop before the stage of microalbuminuria [14, 17, 22, 23, 26, 31, 32]. The urinary NGAL level (1.5-fold) already was significantly elevated in normoalbuminuric patients with diabetes compared with nondiabetic controls and was significantly associated with albuminuria [14]. Mean urinary NGAL and NGAL/creatinine ratio (NGAL/Cr) levels in both microalbuminuric and nonmicroalbuminuric diabetic patients were found to be higher than those in the controls, indicating that tubular involvement may precede glomerular involvement, as urinary NGAL levels are increased in the very early phase of diabetes before microalbuminuria develops [24].

The increased levels of urinary NGAL in diabetic patients, with or without albuminuria, point to early tubular damage and can be used as an early sensitive marker in detecting DN [14, 22, 27, 31]. In addition, urinary NGAL levels were increased in diabetic patients with normal or mildly increased albuminuria, which indicated that tubular and glomerular injuries may be occurring even at the earliest stage of diabetic kidney disease and urinary NGAL could be an early marker of renal dysfunction in diabetic patients without current evidence of nephropathy [19, 31].

NGAL increases in diabetic patients, even before diagnosis of microalbuminuria representing an early marker of “normoalbuminuric” DN, and could be used for the evaluation of early renal involvement in the course of diabetes [17]. Urine NGAL was significantly increased in diabetic patients, even normoalbuminuric, and was positively correlated with HbA1c, duration of diabetes, and urine ACR, suggesting that urinary NGAL could have the potential to be an earlier marker of DN, in normoalbuminuric patients, as a supplement to albuminuria [23]. In addition, urinary levels of NGAL were higher in normoalbuminuric patients with diabetes than in control subjects and increased with increasing categories of albuminuria [14, 29].

Because levels of urinary NGAL were significantly different according to the degree of albuminuria and increased in parallel with the severity of renal disease, reaching higher levels in patients with manifest DN, urinary NGAL levels expresses the degree of renal impairment in DN and, together with albuminuria, it could be used as a sensitive and specific markers for predicting the progression of DN [14, 17, 19, 22, 26, 31]. Furthermore, baseline levels of urinary NGAL were significantly elevated and correlated with the severity of albuminuria in patients with diabetes and were observed to be significantly correlated with a rapid decline in the eGFR [33]. Elevated levels of urinary NGAL are also predictive of decline in eGFR in type 2 diabetic patients with micro- or macroalbuminuria [34]. Another study also found that urinary concentrations of NGAL were associated with progression to ESRD and death in type 2 diabetes, even after adjustment for baseline albuminuria and GFR, indicating the potential importance of urinary NGAL for the identification of persons most likely to progress to ESRD or to premature death [35].

## 3. Kidney Injury Molecule-1

Kidney injury molecule-1 (KIM-1) is a transmembrane protein and its expression is not measurable in normal proximal tubule cells but is markedly upregulated with injury/dedifferentiation [36]. It has been suggested that its presence in the urine is highly specific for kidney injury and may serve as a useful biomarker for renal proximal tubule injury facilitating the early diagnosis of the disease [36, 37]. Renal tubular damage, as evidenced by increased levels of urinary KIM-1, is evident even prior to the development of diabetes and overt kidney disease [38].

Urinary levels of KIM-1 were significantly higher in patients with diabetes when compared with nondiabetic control subjects and correlated with urinary albumin, UACR, SCr, blood urea, and BUN, indicating the possible clinical application of urinary KIM-1 as a complementary marker for early detection of DN [14, 18, 21, 22, 27, 39]. Urinary KIM-1 levels have also been found to be correlated with the urinary IL-18 and angiotensinogen, body mass index, duration of diabetes, glycemic control, and systolic and diastolic blood pressures (BP), which may reflect the role of Kim-1 as a marker for diagnosis and prognosis of DN among diabetic patients taking into account other risk factors [19, 21, 39–41]. In addition, urinary KIM-1 levels were significantly increased both in subgroups of DN and in chronic kidney disease (CKD) compared with controls [41]. In this study, urinary KIM-1 levels, along with urinary albumin excretion and the duration of diabetes, were found to be independent risk factors associated with low GFRs [41].

Urinary levels of KIM-1 were found significantly elevated in diabetic patients with microalbuminuria, in comparison with diabetics with normoalbuminuria and nondiabetic healthy controls, demonstrating the existence of diabetic tubular damage at the early stage of DN [16, 18, 21, 39, 41]. In one study, urinary Kim-1 levels were elevated significantly (10-fold) in type 2 diabetic microalbuminuric patients (whose SCr level was <2 mg/dL) as compared to the controls and normoalbuminuric patients, indicating its potential value in the identification of diabetics with nephropathy at the early stage [39]. Urinary KIM-1 was also significantly increased in early detected inflamed kidney of diabetic patients compared to controls and was positively associated with degree of kidney inflammation [42]. The highest level of KIM-1 was found in the DN patients compared to the kidney inflammation state alone, suggesting that urinary KIM-1 excretion could help differentiate kidney inflammation versus DN [42]. In addition, urinary KIM-1 levels were increased in type 2 diabetic patients with normal or mildly increased albuminuria, indicating that tubular and glomerular injuries may coexist at the earliest stage of diabetic kidney disease and KIM-1 could be potential marker of early DN [19].

Urinary KIM-1 excretion is elevated in diabetic patients compared to controls, even before they develop microalbuminuria, indicating that diabetic tubular involvement may precede glomerular involvement and, by measuring KIM-1, this “tubular phase” of renal damage could be detected before albuminuria becomes pathologically elevated [14, 16, 18, 22, 32, 41]. Urinary KIM-1 levels seems to predict renal injury secondary to DN in early period independent of albuminuria, because urinary KIM-1 was elevated despite normal urinary albumin excretion in the normoalbuminuric subgroup [41]. It has been suggested that increased renal biomarkers, such as urinary KIM-1 in diabetics, are early sensitive and specific markers of DN, even preceding the development of microalbuminuria, denoting that they can be used as early and sensitive markers for early detection of DN [18].

In type 2 diabetes, urinary KIM-1 was markedly increased compared with the controls and its levels increased from the normoalbuminuria to the last macroalbuminuria group, predicting the progression of DN [27]. Urinary Kim-1 also was high in normoalbuminuric diabetics before reduction in GFR, indicating early diabetic kidney injury [32]. Furthermore, higher levels of urinary KIM-1 were associated with a faster decline in kidney function and an increased risk of mortality during 4 years of follow-up [40]. Another follow-up study among type 2 diabetic patients with various degrees of incipient or established DN also found that higher levels of urinary KIM-1 were associated with a faster decline in eGFR [34]. Moreover, low baseline levels of urinary KIM-1 were strongly associated with regression of microalbuminuria independent of clinical characteristics [16]. In another study, Irbesartan treatment significantly reduced levels of the tubular marker urinary KIM-1 in patients with type 2 diabetes and microalbuminuria, indicating the role of KIM-1 in monitoring treatment effect in DN [43].

## 4. Liver-Type Fatty Acid Binding Protein

Liver-type fatty acid binding protein (L-FABP) is a low molecular weight (15 kDa) intracellular carrier protein that is expressed in the renal proximal tubule and liver. In renal disease L-FABP gene expression in the kidney was upregulated and its urinary excretion was found to correlate with the severity of tubulointerstitial injury, reflecting stresses on the proximal tubules [44]. A study in CKD showed that serum L-FABP levels do not influence the urinary L-FABP level, which suggested that the measured L-FABP in urine originates primarily from tubular cells [45]. Urinary L-FABP was significantly higher in diabetic patients compared with healthy controls and correlated with albumin excretion rate and creatinine clearance (CrCl) [46]. Urinary L-FABP levels were significantly higher in diabetic patients with nephropathy than in healthy subjects and correlated positively with urinary albumin, UACR, and albuminuria and inversely with GFR, indicating its possible clinical application as a complementary marker of DN [47–49].

Urinary L-FABP levels were significantly increased in macroalbuminuric patients compared with microalbuminuric and currently normoalbuminuric patients [46]. In one study from type 2 diabetes mellitus patients, elevated levels of urinary L-FABP were evident from the microalbuminuric stage, indicating tubular damage at the early stage of DN [50]. According to this study, urinary excretion of L-FABP levels in the microalbuminuric group was significantly correlated with systolic BP, fasting plasma glucose, and HbA1c, which indicated that urinary L-FABP could be most sensitive marker for detecting glomerular and tubular dysfunction at the early stage of DN [50].

Additionally, urinary levels of L-FABP in patients with microalbuminuria were significantly higher than in those with normoalbuminuria [18, 47, 51]. Increased urinary L-FABP excretion has been also found in the micro- and macroalbuminuric patients compared with the patients with persistent normoalbuminuria [52]. Urinary L-FABP levels were also elevated in patients with reduced eGFR and showed a positive correlation with systolic BP and protein/Cr ratio, suggesting the importance of tubular damage in the development of DN and urinary L-FABP excretion in the assessment of tubular dysfunction in early DN [50].

Urinary L-FABP excretion is higher in diabetic patients compared to healthy controls, including in patients without current evidence of nephropathy (normoalbuminuria), indicating its value in detecting DN even before the appearance of pathological albuminuria, the earlier measurable sign of renal diabetic involvement [18, 48, 53, 54]. Urinary levels of L-FABP were significantly higher in the patients with type 2 diabetes who had normoalbuminuria than in normal control subjects and progressively increased in subjects with normo-, micro-, or macroalbuminuria and further increased in patients with ESRD [54]. The levels of urinary L-FABP in each DN group were significantly different from the levels in all of the other groups and significantly increased according to the severity of DN [47, 49, 54]. Levels of urinary L-FABP were elevated in normoalbuminuric patients than in the controls and were further increased with increasing levels of albuminuria, indicating its value in accurately reflecting severity of tubular damage in the early stage of DN [48]. In the prospective study, high urinary L-FABP levels were associated with the increase in albuminuria, progression to ESRD, or induction of hemodialysis [54]. Surprisingly, even in the subgroup of patients without renal dysfunction, higher urinary levels of L-FABP were associated with the progression of DN, demonstrating the usefulness of urinary L-FABP as a marker for predicting the progression of DN in the early stage [54].

Urinary L-FABP was elevated at an early stage, even before any clinical signs of glomerular damage are detectable, and independently predicted the development of microalbuminuria and death, suggesting its value as a useful marker for the detection of tubular damage early in the course of diabetes and for the prediction of DN and death [52]. In addition, urinary concentrations of L-FABP were associated with progress to ESRD in patients with type 2 diabetes, even after adjustment for baseline albuminuria and GFR, indicating the potential importance of urinary L-FABP in identification of persons most likely to progress to ESRD [35]. A long term observational study on type 2 diabetic patients without advanced nephropathy revealed that higher urinary levels of L-FABP were associated with deteriorating renal function and a higher incidence rate of CVD. This association was markedly observed even in patients with normoalbuminuria, which indicated its potential role as a marker for predicting future renal dysfunction and incidence of CVD in diabetic patients with an early stage of nephropathy, independently of albuminuria [51].

## 5. N-Acetyl-beta-D-glucosaminidase

N-acetyl-beta-D-glucosaminidase (NAG) is a hydrolytic lysosomal enzyme found predominantly in proximal tubule. It has been demonstrated as a useful marker of renal tubular impairment in various conditions involved with renal injury or dysfunction [55]. Urinary activities of NAG are elevated in patients with diabetes when compared with nondiabetic control subjects and showed a significant positive correlation with serum Cys C, SCr, UAE, and UACR and an inverse correlation with measured and estimated CrCl in all patients, indicating the possible clinical application of urinary NAG as a complementary marker for early detection of DN [14, 25, 56–60]. It is also correlated positively with disease duration and poor glycemic control (HbA1c) [25, 57, 59, 60]. In diabetic patients with poor metabolic control (HbA1c > 8%), a statistically significant increase in urinary NAG was found compared with the diabetic patients with good metabolic control [25, 60].

In diabetics, urine NAG level was significantly higher in microalbuminuric patients compared to both normoalbuminuric patients and controls, suggesting that tubular dysfunction is already present in this period [16, 25, 56, 57, 59–61]. Urinary NAG excretion was significantly higher in all patients with type 2 diabetes than in controls and in microalbuminuric than in normoalbuminuric patients, representing early marker of incipient DN [25]. The ROC curve analysis of the above study showed that urinary NAG is the most sensitive marker of microalbuminuria and early renal damage with sensitivity of 83.3% and specificity of 77.8% [25].

It was also suggested that NAG had higher sensitivity as urinary marker in early detection of tubular and glomerular lesions in diabetic patients and could be used as screening test for early diagnosis of DN [56]. In another study, urinary NAG and microalbuminuria in the diabetic patients were significantly increased compared to those in the controls and urinary NAG showed the highest sensitivity and specificity (100% and 87.5%, resp.) as compared to sensitivity and specificity of SCr, CrCl, and microalbuminuria (25% and 24.9%, 50% and 58.3%, and 25% and 75%, resp.) [60]. The investigators from this study suggested that measuring urinary NAG excretion could be useful for the assessment of renal failure in patients with diabetes and confirmed the use of this enzyme as a routine screening test [60].

Compared to healthy controls, urinary NAG excretion is higher in diabetic patients, even before they develop microalbuminuria [14, 25, 56, 59, 61–64]. Urinary NAG activity was within the normal ranges in the healthy control groups and significantly increased over the upper reference limit in the groups of patients with normoalbuminuria, indicating the great importance of NAG in discovering the renal tubule cells damage, especially at the early stage before the appearance of microalbuminuria [65]. On the other hand urinary NAG already showed 9-fold increase in the normoalbuminuric patients compared with the controls, whereas albuminuria (and eGFR) in these patients was comparable with controls, which demonstrated the potential value of urinary NAG as sensitive marker of DN as its level increases before other traditional markers become pathologically elevated [14, 62]. Thus, values of the urinary NAG were elevated before microalbuminuria was observed, with the highest values detected in the group of patients with microalbuminuria, indicating that increased excretion of urinary NAG points to early tubular damage and can be used as the most sensitive marker in the early detection of DN [61, 66].

In a more recent study, urinary NAG excretion gradually increases with the increase in duration of diabetes and appeared much before the microalbuminuria, decreased eGFR, and increased SCr. In this study, the urinary NAG activity increased 16- and 18-fold in moderately increased albuminuria and DN patients, respectively, without any change in non-DN patients. A cutoff value of 3 U/L of urinary NAG has demonstrated a sensitivity of 96.1% and a specificity of 100% discriminating healthy controls from patients with microalbuminuria (AUC 0.999) and DN (AUC 1.000), and the investigators concluded that the urinary NAG may be considered as a potential site-specific early tubular damage marker leading to DN [67]. In addition, significantly increased levels of urinary NAG were found in diabetic patients with varying degree of albuminuria and this increase was parallel to the severity of renal involvement expressed with the level of albuminuria [14, 56, 59, 60, 68]. Urinary levels of NAG were higher in patients with diabetes than in controls and increased with increasing categories of albuminuria, suggesting that it could be used as a useful marker reflecting the degree of renal impairment in DN [14, 59].

Urinary NAG activities tended to be higher in diabetic patients with and without albuminuria than in control subjects and differences among the diabetic groups were statistically significant, which implies that this enzyme is a more sensitive marker of tubular damage and could be used as a biomarker for the detection of early stage of DN, even in normoalbuminuric patients. Furthermore, baseline urinary excretion of NAG and rising NAG excretion across time predict both microalbuminuria and macroalbuminuria, which suggested that early NAG excretion may be a marker of susceptibility to DN and combining AER and NAG in repeated measures may help to identify individuals susceptible to DN [69]. Also, low baseline concentrations of urinary NAG were significantly associated with the regression of microalbuminuria over the subsequent 2 years, indicating that tubular dysfunction is a critical component of the early course of DN and urinary NAG can be used as an early marker of normoalbuminuric DN [16].

## 6. Alpha-1 Microglobulin

Alpha-1 microglobulin (A1M) is a small molecular weight protein (27 kDa) present in various body fluids. In the healthy kidney, it passes freely through the glomerular membranes, and about 99% is reabsorbed and catabolized by the proximal tubular cells. Increased A1M in urine can therefore be an early sign of renal damage, primarily on the proximal tubules [70]. The level of urinary A1M was significantly increased in the group of diabetic patients as compared to the level of normal subjects [71]. Urinary A1M levels were markedly elevated in diabetic patients when compared with control subjects [61, 72–74] and correlated directly with urinary albumin excretion, UACR, and serum CysC and negatively with eGFR [72–76], indicating the possible clinical application of urinary A1M as a complementary marker for early detection of DN. It also correlates with urinary advanced glycation end-products, diabetes duration, HbA1c, fasting, and postprandial blood glucose [72, 73]. Urinary A1M level was higher in the patients with poor glucose control (HbA1c > 8.5%) and directly related to albuminuria [61]. Urinary A1M was also related to the duration, severity, and control of diabetes, indicating that it is a good marker of the severity of renal impairment in type 2 diabetic subjects [75].

A proteomic based study among microalbuminuric diabetic patients showed the early and coappearance of A1M with albumin, demonstrating that urinary A1M can be used as markers for specific and accurate clinical analysis of DN [77]. The concentration of A1M during the development of albuminuria also showed a very strong positive correlation [61]. In addition, the urinary excretion of A1M was significantly higher in microalbuminuric in comparison with normoalbuminuric patients and controls, indicating tubular damage at an early stage of DN [61, 72, 78]. It also demonstrated the importance of tubular dysfunction, as an early and integral component of the DN in diabetic patients [73]. A1M is a marker of tubular dysfunction in diabetic patients and hyperglycemia was the most important risk factor associated with A1M urinary excretion, emphasizing the value of tight glycemic control in slowing the progress of tubular dysfunction in diabetic patients [73, 79].

In a sample of community treated type 2 diabetic subjects, 45.2% had elevated A1M urinary excretion, 32.7% had micro/macroalbuminuria, and 27.2% had a GFR < 60 mL/min, indicating tubular dysfunction and nonalbuminuric renal disease in patients with diabetes [79]. In addition to albuminuria measuring glomerular dysfunction, urinary A1M estimating proximal tubular dysfunction is useful for the early detection of nephropathy in diabetes [75]. It was also suggested that urinary A1M provides a noninvasive and inexpensive diagnostic alternative for the early detection of tubular disorders of DN [71].

Urinary A1M is a sensitive biomarker in detecting tubular dysfunction in early DN, even in normoalbuminuric patients, demonstrating that tubular injury is an early event in diabetes [61, 76, 80–83]. Diabetic patients with normoalbuminuria excreted significantly higher levels of urinary A1M compared with healthy individuals, while there was no significant difference between the patients and controls in respect to serum/urine Cr [61]. In another study, urinary A1M was increased in 27.9% normoalbuminuric type 2 diabetic patients, indicating that urinary A1M precedes the onset of albuminuria and may serve as a marker in early DN [80]. Elevated levels of urinary A1M in normoalbuminuric patients with diabetes showed that proximal tubule dysfunction may develop before the stage of microalbuminuria and that A1M is a significant biomarker for incipient DN [81]. A1M also correctly identified normoalbuminuric diabetics from healthy controls with accuracy of 89.0%, sensitivity of 86.3%, and specificity of 94.2% [83]. Urinary A1M levels also significantly increased with severity of albuminuria, indicating its value in predicting progression of DN as suggested by increasing albuminuria [75].

## 7. Beta 2-Microglobulin

Beta (*β*) 2-microglobulin (B2-M) is a low molecular weight protein (11.8 kDA), produced by all cells expressing major histocompatibility complex class I antigen. It is readily filtered through the glomerulus and almost completely reabsorbed and catabolized by the renal proximal tubules. Increase in urinary B2-M indicates tubular dysfunction, and measurement of B2-M in urine is a sensitive and reliable assay for detecting tubular injury [84]. The level of B2-M in urine of the patients with diabetes was higher than normal [85, 86] and showed significant correlation with urinary albumin excretion and 2-hour postprandial blood sugar [85]. Urine B2-M in the children with type 1 diabetes was significantly increased compared to the controls and correlated positively with disease duration and glycemic control [87].

In type 2 diabetes, significant correlation was found between level of microalbumin and urine B2-M with length of diabetes and serum and urine Cr [86]. Urinary B2-M excretion is elevated in the patients with a poor metabolic control (HbA1c > 8.0%) compared to those with a good one [61]. Urinary B2-M levels were higher in diabetic patients with macro- and/or microvascular complications, indicating that increased urinary B2-M excretion was associated with more severe disease in these patients [85, 88]. In addition, they appear to be useful in early detection of DN with positive correlation with the duration of type 1 diabetes and glycemic control (HbA1c) [87]. Most importantly, urine B2-M was able to reliably identify biopsy-proven DN, indicating the potential clinical application of urinary B2-M as a marker for early DN in diabetic patients [89].

Urinary B2-M exhibited a significant positive correlation with urinary albumin levels and negative correlation with GFR and serum B2-M in type 2 DN [90]. This study also found that diabetic kidney damage exhibits an initial increase in urinary B2-M levels, as compared with nondiabetic kidney damage, and renal dysfunction aggravated as urinary B2-M levels gradually increased [90]. In addition, urinary B2-M excretion is significantly higher in the patients with microalbuminuria than normoalbuminuria and in the controls, indicating the presence of tubular injury in early DN as characterised by increased B2-M excretion [61, 78, 85]. In patients with type 2 diabetes and biopsy-proven DN, urinary excretion of B2-M was significantly correlated with the severity of tubulointerstitial injury, demonstrating the usefulness of B2-M as marker of tubular dysfunction in early DN [91]. A urinary proteomic analysis study found high amounts of B2-M in the urine of diabetic patients with macro- or microalbuminuria compared with controls and patients without micro- or macroalbuminuria [92].

Increased excretion of B2-M was found in early course while albumin excretion was still in normal range in the urine of diabetic patients, which indicated that the increase in urinary B2-M precedes the stage of albuminuria and that early DN is related to proximal tubule dysfunction [80–82]. Urinary B2-M was increased in 23.5% of normoalbuminuric patients with type 2 diabetes, suggesting that proximal tubule dysfunction may be responsible for early DN independently of preceding glomerular endothelial dysfunction and urinary B2-M may be used as sensitive marker in the diagnosis of early DN [80]. In addition, the urinary excretion of B2-M increased progressively from normoalbuminuria to macroalbuminuria, indicating its value in predicting progression of DN at early stage [85].

## 8. Retinol Binding Protein

Retinol binding protein (RBP) is another low molecular weight protein (21 kDa) which is freely filtered at the glomerulus and then almost completely reabsorbed in the proximal tubule. Both serum and urine levels have been shown to be elevated in patients with diabetes [85, 93, 94]. RBP showed significant positive correlations with triglyceride, systolic BP, and log urinary albumin excretion [93]. Urinary RBP excretion has been found to be increased in diabetic subjects compared with healthy controls [87, 95–97] and to correlate with UAE, serum and urine Cr, CrCl, and 24 h urine protein, indicating its potential clinical application as a marker of early DN [85, 95, 97]. It has been also shown to correlate closely with duration of diabetes and glycemic control (HbA1c) [87, 94, 95, 97].

Urinary RBP4 levels were higher in subjects with prediabetes or type 2 diabetes than in subjects with normal glucose tolerance and correlated strongly with fasting glucose, triglycerides, BP, eGFR, and UACR [98]. In addition, urinary excretion of RBP was higher in patients with macro- and/or microvascular complications of diabetes compared to those without, which confirmed the utility of RBP as a renal biomarker for predicting diabetic complications [85, 88]. Urinary RBP was also a predictor of the risk of dialysis, doubling of SCr, or death in diabetic patients with macroalbuminuric DN, suggesting that RBP may serve as a marker to follow-up clinical monitoring of diabetics with DN [99].

Levels of urinary RBP were significantly higher in microalbuminuric diabetics when compared with normoalbuminuric and normal controls, indicating impaired proximal renal tubular function in early stage of DN [85, 95, 97]. In one study, diabetic patients with microalbuminuria had concomitant renal tubular disorder indicated by high urinary RBP in 90.9% of them, which suggested that elevated urinary RBP might be a useful marker of renal injury in early DN [95]. Furthermore, urinary RBP4 was highly predictive of microalbuminuria, even after adjustment for other metabolic parameters. In this study, urinary RBP4 concentration showed a stronger association with urinary ACR than serum RBP4 concentration for microalbuminuria and combined micro- and macroalbuminuria. The AUC (diagnostic accuracy) for urinary RBP4 to detect the presence of microalbuminuria was 0.80 ± 0.02 with sensitivity of 80.18% and specificity of 64.03%; urinary RBP4 may therefore be used as early stage marker for predicting of diabetic renal damage [98].

Urinary RBP excretion is higher in diabetic patients compared to healthy controls, even before the diagnosis of microalbuminuria [95, 96]. In the above study, urinary RBP excretion was significantly higher in normoalbuminuric patients than controls and showed a significant correlation with urinary NAG and HbA1c in these patients [95]. Among these normoalbuminuric patients, 82% had raised urinary excretion of RBP, which suggested that proximal tubular dysfunction may occur independently of glomerular alteration [95]. In normoalbuminuric diabetics, the excretion rate of RBP was significantly higher compared to control subjects and correlated to the excretion rate of NAG and albumin [100].

Among type 2 diabetic patients, 50% were positive for urinary RBP, while 28% and 6% of them were positive for micro- and macroalbuminuria, respectively [85]. The increase in the urinary excretion of RBP4 in diabetics is highly specific for tubular disease, which occurs earlier than glomerular (albumin) affection, as urinary RBP4 excretion is increased in early DN and might even be a marker of early renal damage preceding microalbuminuria [96]. Furthermore, the urinary excretion of RBP increased progressively from normoalbuminuria to macroalbuminuria, indicating progression of DN at the early stage [85].

## 9. Conclusions

Tubular injury, as shown by increased urinary tubular damage markers at the microalbuminuria stage of diabetes, is a critical component of the early course of DN. Urinary excretion of these tubular markers is significantly higher in diabetics compared to healthy controls, even before the diagnosis of microalbuminuria, supporting the hypotheses that tubular injury is an early event in diabetes. The tubular markers discriminate between healthy subjects and diabetics in early stages of nephropathy and might also serve as a marker of the efficacy of renal protective agents. Urinary markers of tubular injury are early, sensitive, and specific markers of DN, even preceding the development of microalbuminuria, denoting that they can be used as early and sensitive markers for early detection of DN. Despite the promise of these new tubular injury markers, further large, multicenter prospective studies are still needed to confirm their clinical utility as urinary markers in early DN for everyday practice.

## Abbreviations

A1M: Alpha- (*α*-) 1 microglobulin
B2-M: Beta (*β*) 2-microglobulin
DN: Diabetic nephropathy
eGFR: Estimated glomerular filtration rate
KIM-1: Kidney injury molecule-1
L-FABP: Liver-type fatty acid binding protein
NAG: N-Acetyl-beta-glucosaminidase
NGAL: Neutrophil gelatinase associated lipocalin
RBP: Retinol binding protein
UACR: Urinary albumin-creatinine ratio.
